# Supplementary material for: Exploring caregivers’ experiences of Kangaroo Mother Care in Bangladesh: A descriptive qualitative study
Source: PLoS One. 2023 Jan 23;18(1):e0280254. doi: 10.1371/journal.pone.0280254 (PMC9870098; doi:10.1371/journal.pone.0280254)
Supplement: S1 Appendix — (DOCX) [file pone.0280254.s001.docx]

# S1 Appendix. According to COREQ 32-item checklist

Tong A, Sainsbury P, Craig, J. Consolidated criteria for reporting qualitative research (COREQ): a 32-item checklist for interviews and focus groups. Int J Qual Health Care. 2001;19(6): 349-357. doi:10.1093/intqhc/mzm042.

| **No.** | **Item** | **Guide**  **questions/descriptions** | **Reported on page** |
| --- | --- | --- | --- |
|  | Domain1:  Research team and reflexivity | | |
|  | **Personal Characteristics** | | |
| 1. | Interviewers/facilitators | Which author/s conducted the interview or focus group? | The first two authors (JS) and (HO) conducted the interviews together with a data collection team. |
| 2. | Credentials | What were the researcher’s credentials? | (JS) is a pediatric RN and holds a MNSc, Uppsala University. (HO) was a medical student at the time for the data collection, Uppsala University (GB) is a medical doctor at icddr,b, (AER is a medical doctor) at icddr,b (YTB) RN, PhD, Uppsala University (SMR) a medical doctor, PhD Uppsala University (MM) a medical doctor, PhD and professor in Global Health, Uppsala University |
| 3. | Occupation | What was their occupation at the time of the study? | (JS) works as a teacher in the nursingprogramme and is a pediatric nurse at Childrens´Hospital in Uppsala, (HO) was a medical student, Uppsala University.( GB) a researcher at icddr,b. (AER) a researcher at icddr,b., (YTB) head nurse at NICU and associate professor Childrens´Hospital innUppsala. (SMR) researcher and teacher, Uppsala University. (MM) professor in Global Health, Uppsala University |
| 4. | Gender | Was the researcher male or female? | (JS) female, (HO) female (AER) male, (YTB) female, (SMR) male, (MM) male |
| 5. | Experience and training | What experience or training did the researcher have? | (JS) has previous training in qualitative methods and experience in conducting interviews  (HO) has training in qualitative methods and interview methodology. (YTB) is an experienced qualitative researcher |
|  | **Relationship with participants** | | |
| 6. | Relationship established | Was a relationship established prior to study commencement? | No, the researchers did not know or meet any of the participants. |
| 7. | Participant knowledge of the interviewer | What did the participants know about the researcher/s? | Oral information to the participants about the study’s aim, design, and research questions as well about the researcher. |
| 8. | Interviewer characteristics | What characteristics were reported about the interviewer/facilitator? | Participants were informed about the researchers’ credentials, occupations, and clinical experience; additionally, how the interviews were planned. |
|  | Domain 2:  study design | | |
|  | **Theoretical framework** | | |
| 9. | Methodological orientation and Theory | What methodological orientation was stated to underpin the study? | We used thematical analysis. |
|  | **Participant selection** | | |
| 10. | Sampling | How were participants selected? | Convenience sampling was used among willing caregivers to participate in the interview during the data collection period. Consent was required to be included in the study. |
| 11. | Method of approach | How were participants approached? | The caregivers were asked to participate by the nurses at the ward. All caregivers requested agreed to participate in the study. |
| 12. | Sample size | How many participants were in the study? | Ten caregivers |
| 13. | Non‐participation | How many people refused to participate or dropped out? Reasons? | All caregivers requested agreed to participate in the study. |
|  | **Setting** | | |
| 14. | Setting of data collection | Where was the data collected? | Data were collected in the KMC wards, in a separate room when the caregiver came back to the facility for the one-week follow-up or in the home of the caregiver. |
| 15. | Presence of non-participants | Was anyone else present besides the participants and researchers? | Yes, other caregivers were present in the room and the data collection team. |
| 16. | Description of sample | What are the important characteristics of the sample? | The study participants were caregivers who had experience of performing KMC. |
|  | **Data collection** | | |
| 17. | Interview guide | Were questions, prompts, guides provided by the authors? Was it pilot-tested? | Yes, we used a semi-structured interview guide (Supplementary file S2 and S3).  The interview guide was pilot-tested. |
| 18. | Repeat interviews | Were repeat interviews carried out? | Yes, one |
| 19. | Audio/visual recording | Did the research use audio or visual recordings to collect the data? | The interviews were conducted face to face in Bangla and were audio recorded. |
| 20. | Fieldnotes | Were field notes made during and/or after the interview? | Yes, field notes were taken by (JS) and (HO) and were used to ensure the details of the transcripts, including non-verbal communication. |
| 21. | Duration | What was the duration of the interviews | 16-47 minutes |
| 22. | Data saturation | Was data saturation discussed? | Yes |
| 23. | Transcripts returned | Were transcripts returned to participants for comment and/or correction? | No |
|  | Domain 3:  analysis and findings | | |
|  | **Data analysis** | | |
| 24. | Number of data coders | How many data coders coded the data? | JS and HO |
| 25. | Description of the coding tree | Did the authors provide a description of the coding tree? | Yes, see table 1 with Theme and subthemes |
| 26. | Derivation of themes | Were themes identified in advance or derived from the data? | They were derived from the data – inductive. |
| 27. | Software | What software, if applicable, was used to manage the data? | World and excel were used in sorting the data |
| 28. | Participant checking | Did participants provide feedback on the findings? | No |
|  | **Reporting** | | |
| 29. | Quotations presented | Were participant quotations presented to illustrate the themes/findings? Was each quotation identified? | Yes |
| 30. | Data and findings consistent | Was there consistency between the data presented and the findings? | Yes |
| 31. | Clarity of major themes | Were major themes clearly presented in the findings? | Yes |
| 32. | Clarity of minor themes | Is there a description of diverse cases or discussion of minor themes? | Yes |
