# Supplementary material for: Exploring caregivers’ experiences of Kangaroo Mother Care in Bangladesh: A descriptive qualitative study
Source: PLoS One. 2023 Jan 23;18(1):e0280254. doi: 10.1371/journal.pone.0280254 (PMC9870098; doi:10.1371/journal.pone.0280254)
Supplement: S2 Appendix — (DOCX) [file pone.0280254.s002.docx]

**S2 Appendix. Interview guide for in-ward interviews**

**Interview guide for questions to mothers/caregivers in-ward interviews (English)**

**Inform about the aim of the interview**

The aim with the interview is to explore and describe the parents/caregivers experience of the Kangaroo Mother Care with a focus on enablers and barriers

- Introduction of principal investigator and interviewer
- Inform about confidentiality and volontary participation
- Signing of consent form
- Background information: see separate form age, education, number of children, days for the infant admitted in the hospital, infants gestational age, weight at birth, who is the performing STS

**Supplementary questions/in-depth questions**

- Tell me more…
- How was it when…
- How did you percieve…
- Can you tell me more about how you felt

**Icebreaker question**

What is you name? How are you? For how long have you performed KMC? How is your baby?

**General question**

What do you think of Kangaroo Mother Care? How do you define KMC? What are the benefits of KMC?

**What is your name? How are you? For how long have you performed KMC? How is your baby? Specific questions**

1. Do you have any idea regarding skin to skin care, position, timing and duration of skin to skin care, how to bind the baby. From whom did you get this information?
2. What support did you get from healthcare staff to perform:

- skin to skin (position, teaching from health about position of skin to skin care, how to sit during skin to care, timing of skin to care, initiation and duration of skin to skin care, help giving when the caregivers goes to toilet, prepare food
- breastfeed/ giving breastmilk? (teaching the caregivers about the position and attachment of breastfeeding, giving breastmilk, or extracting breastmilk, teaching the mother to provide cupfeeding if baby don´t suck , initiation and duration of breastfeeding or cup/ nasogastric tube
- What support/facilitation did you get from your family to perform skin to skin care in the facility? (coming with prepared meals for mother, taking care of siblings, assist skin to skin)

1. What motivates you to perform skin to skin/breastfeeding/giving breastmilk?
2. What are the hindrance for you to perform skin to skin/breastfeeding/giving breastmilk and continue KMC at facility?

- Feeling discomfort in providing skin to skin care
- Privacy
- Behavior and availability of healthcare staff
- Response from health care staff, did they listen to you?
- Support from family members (mother in law, husband, other family members
- Society and culture support to perform skin to skin?

1. How can we overcome this hindrance: discomfort, privacy, healthcare staffs behaviour, support from family member, society and cultural support?
2. Are you satisfied regarding the KMC service in the health facility?
3. What can facilitate you to perform the KMC in facility

- How long should you continue KMC at home after discharge?
- What can be the hindrance for you to continue KMC at home
- Wat can facilitate you to perform KMC at home

1. Do you when you need to bring back/seek care for your baby to the hospital (danger signs)/
2. Do you know about the follow up visits after discharge. If yes, how many follow up visits and when?
3. Why is the follow up visits important for the baby?
4. Do you have any recommendation of how the quality of KMC can be improved?

Thank you for your participation!
