# Supplementary material for: Exploring caregivers’ experiences of Kangaroo Mother Care in Bangladesh: A descriptive qualitative study
Source: PLoS One. 2023 Jan 23;18(1):e0280254. doi: 10.1371/journal.pone.0280254 (PMC9870098; doi:10.1371/journal.pone.0280254)
Supplement: S3 Appendix — (DOCX) [file pone.0280254.s003.docx]

**S3 Appendix. Interview guide for follow-up interviews**

**Interview guide for questions to mothers/caregivers follow-up interviews (English)**

**Inform about the aim of the interview**

The aim with the interview is to explore and describe the parents/caregivers experience of caring for the low birth weight baby and the use of ThermoSpot for this purpose.

- Introduction of principal investigator and interviewer
- Inform about confidentiality and voluntary participation
- Signing of consent form
- Background information: age, education, number of children, days for the infant admitted in the hospital, infants gestational age, weight at birth, who is the caregiver performing KMC

**Initial question**

Can you tell me about your experience of caring for your newborn? What has been difficult? What has worked well?

**Larger conversations areas**

1. How did the care for your newborn work out at the hospital? What kind of support did you get? What was difficult?

Follow-up:

- Who gave instructions for the care of the newborn, if any?
- Who participated in the care of the newborn?
- How did you manage the routines? Any aides used?
- What do you wish would have been different? Any additional help, instructions, etc

1. How did the care for your newborn work out when you came home? What kind of support did you get? What was difficult?

Follow-up:

- Was there any difference to care for the baby at home compared to when you were at hospital? If so, in what way?
- Who participated in the care of the newborn at home?
- How did you manage the routines? Any aides used?
- What do you wish would have been different? Any additional help, instructions, etc

1. Did you experience any occasions, either at hospital or at home, when your baby needed extra care or attention? Like being too cold, having a fever, crying excessively or similar? If so, can you please tell me what happened?

Follow-up:

- What did you do to solve the problem?
- Did you encounter any problems when trying to solve the problem?
- Who helped you, if anyone?

1. At the hospital you were introduced to ThermoSpot as a help to see if your baby is not getting cold. Can you tell me about your experiences of this ThermoSpot?

Follow-up:

- Did you use to look at the ThermoSpot? If so, when and how often? Was it helpful? If so, in what way?
- If you did not use the ThermoSpot, can you please tell me why?
- Who introduced you to the ThermoSpot? Did you get any support or encouragement to use it?
- Have you continued to use ThermoSpot at home? If not, why?

**Keywords/Themes important to discuss:**

- Your role as parent/caregiver
- Parents/caregivers presence and participation
- Communication between healthcare provider and you as parent/ caregiver
- Needs as parent/caregiver
- Wishes as parent/caregiver
- Satisfaction as parent/caregiver (KMC and ThermoSpot)
- Any difficulties performing KMC at home?
- Usefulness of ThermoSpot at home?
- Handling of the ThermoSpot fall off?
- Any side effects of ThermoSpot? Rashes, other allergic reactions?
- Anything else to address?

**Supplementary questions/in-depth questions to the above**

- Tell me more…
- Please give example…
- How did you perceive…
- Can you tell me more about how you felt…

Thank you for your participation!
