# Supplementary material for: Exploring caregivers’ experiences of Kangaroo Mother Care in Bangladesh: A descriptive qualitative study
Source: PLoS One. 2023 Jan 23;18(1):e0280254. doi: 10.1371/journal.pone.0280254 (PMC9870098; doi:10.1371/journal.pone.0280254)
Supplement: S4 Appendix — (DOCX) [file pone.0280254.s004.docx]

**S4 Appendix. Consent form for in-ward interviews**

**Consent Form for Interview of Mothers/care givers/family members (English)**

**Protocol Title: Experiences of Kangaroo Mother Care in Bangladesh with focus on enablers and barriers**

**Principal Investigator:** Dr. Ahmed Ehsanur Rahman and Johanna Sjömar

**Organization:** International Centre for Diarrhoeal Disease Research, Bangladesh (icddr,b), and Department of Women´s and Childrens´ Health, Uppsala University, Sweden

**Purpose of the research:**

The purpose of the study is to explore experiences of Kangaroo Mother Care among healthcare provider, community workers and parents/caregivers in Bangladesh Moreover, we want to know about the implementation challenges of KMC and will try to find out ways of overcoming those challenges. We will use these information for the betterment of health care service.

**Why are you invited to participate in this study?:**

You have been admitted to the hospital together with your infant during performing KMC. Your participation in this study will facilitate our understanding about enabling factors and barriers regarding KMC in Bangladesh.

**Methods and Procedure:**

If you agree to our proposal of including you to participate in the study, I will ask you some questions as per our direction. This discussion may require 40-60 minutes. If needed, we will come again for further data collection. For transcription and future use of data, we will use a digital voice recorder to record the interview.

**Risk and Benefits:**

There are no significant risks to participation in the study. We will only collect above mentioned information, for which you will only need to spend some time.

The study may not benefit you directly. The information that we will obtain from this research would have broader impact, guiding of the development of the newborn health and give opportunity of getting benefits of your community.

**Privacy, anonymity and confidentiality:**

Information that you will share with us will remain confidential, under lock and key. None other than the investigators of this research; possible study monitor, and any law-enforcing agency in the event of necessity would have an access to the information. Any personal identifiable information will be held and processed under secured conditions, with access to limited appropriate staff of that organization.

**Future use of information:**

Information provided by you will be a great use for the government and NGOs for developing an effective mechanism for introducing KMC services in Bangladesh.

**Right not to participate and withdraw:**

Your participation in this study is completely voluntary. You have the right to withdraw from the study at any time. Even if you do not agree to join the study, or if you withdraw from the study, you will still receive the same quality of medical care available to you through ICDDR, B, or the government health centres.

**Principle of compensation:**

As we mentioned that you will not be paid or will not get any compensation for participation in the study. Your participation is totally voluntarily.

**Answering your questions/ Contact persons:**

If you have questions about this study or if you feel that you have been treated unfairly or have been hurt by joining the study, you may call Dr. Ahmed Ehsanur Rahman at Centre for Child and Adolescent Health at 8810115 at icddr,b, and Dhaka, Bangladesh. You may also call IRB secretariat, Research Administration, Mr. M A Salam Khan, Phone No: 9886498 or PABX 8860523-32 Extension. 3206.

If you agree to our proposal of enrolling you/your patient in our study, please indicate that by putting your signature or your left thumb impression at the specified space below

Thank you for your cooperation.

**Participant’s Statement:**

Below my signature indicates that I have understand the purpose of the research. I have got all answers about its’ risk and benefits. I have participated in this research willingly and I have informed that to whom I will contact if I have any question or complain. I know that for evidence, one copy of this consent form will be given to me.

Thank you for your cooperation.

_______________________________________ ____________________

Signature or left thumb impression of participant Date

/ Guardian / Attendant

_______________________________________ ___________________

Signature of the PI or his/her representative Date
