# Supplementary material for: Exploring caregivers’ experiences of Kangaroo Mother Care in Bangladesh: A descriptive qualitative study
Source: PLoS One. 2023 Jan 23;18(1):e0280254. doi: 10.1371/journal.pone.0280254 (PMC9870098; doi:10.1371/journal.pone.0280254)
Supplement: Attachment — Submitted filename: Respons to Reviewers.docx [file pone.0280254.s006.docx]

Manuscript Number PONE D-22-08128

Response to Rewiewers, PLOS ONE

The authors would like to thank the reviewers for their input and highly valuable comments for the article titled “Exploring caregivers´ experiences of Kangaroo Mother Care in Bangladesh- a qualitative study”. We believe that the review has resulted in an improved manuscript. Point-to-point answers follow below.

Revised text in the new version of our manuscript is marked in red.

An unmarked version of our revised paper without tracked changes has been upload as a separate file labeled ”Manuscript”.

Journal Requirements

Respons:

1. Thank you, we have made sure the manuscript meets PLOS ONE´s style requirements, including those for file naming.
2. Regarding the minimal dataset underlying the result in the manuscript: All relevant data are within the manuscript and its Supporting Information files. Raw data (i.e. transcripts and translations) cannot be shared publicly due to assurances of confidentiality given to participants at the time of consenting. icddr,b is the local collaborator and ethics approval was obtained from the Ethical Review Committee at icddr,b in Dhaka, Bangladesh. Because of the statutory requirements, internal data policies and regulations existing in the collaborating bodies along with the over-arching General Data Protection Regulation (GDPR), the data must be stored in institutional repository (storage platforms) and cannot be made directly accessible without a review of the request for access to data. Therefore, the data can be accessed only upon formal request that details the purpose of such request. The request will then be processed by the Data Repository Committee (DRC) at icddr,b. Any such request should be directed to the principal investigator Dr. Ahmed Ehsanur Rahman (email: [ehsanur@icddrb.org)](mailto:ehsanur@icddrb.org)) or to the Mr. M A Salam Khan, IRB coordinator secretariat ( email: [salamk@icddrb.org](mailto:salamk@icddrb.org))
3. The manuscript has now the abstract after the title page
4. We have included captions for the Supporting Information files at the end of the manuscript and updated any in-text citations to match accordingly. The reference list has been reviewed to ensure that it is complete and correct.

Additional Editor Comments

Respons:

1. Thank you for the valuable comments. The methodology section in the abstract has been modified and now includes more detail.
2. The discussion has been provided by subheadings related to the themes identified and is discussed accordingly. The discussion has been extended, and changes have been made both in the discussion relating to results and methods.

Reviewers´comments

There were no comments submitted as an attachment file.
