# Supplementary material for: Exploring caregivers’ experiences of Kangaroo Mother Care in Bangladesh: A descriptive qualitative study
Source: PLoS One. 2023 Jan 23;18(1):e0280254. doi: 10.1371/journal.pone.0280254 (PMC9870098; doi:10.1371/journal.pone.0280254)
Supplement: Attachment — Submitted filename: PLOS ONE.docx [file pone.0280254.s008.docx]

**Manuscript Number:** PONE-D-22-08128R1
**Manuscript Title:** Exploring caregivers’ experiences of Kangaroo Mother Care in Bangladesh– a qualitative study.

Thank you very much, Dr. Tai-Heng Chen, Academic editor of the PLOS ONE journal for giving me this opportunity to review this scholarly work which was grounded on a very interesting topic “Exploring caregivers’ experiences of Kangaroo Mother Care in Bangladesh– a qualitative study”.

I would also appreciate the authors of this manuscript for investigating this very important topic and coming up with such interesting findings. I have been impressed with the flow, organization, and way they synthesize the research from beginning to end. All the parts of the manuscript have been clearly stated and narrated. I appreciate the authors for their devoted work. Having this in mind, I do have some questions, comments, and suggestions to be addressed.

General questions:

1. What type of qualitative study it was? Was it phenomenological, Ethnographic, Grounded theory, or ……
2. What were the questions of your research?

**In the methods section**:

In the data collection sub-section;

1. When, and where had the pilot study been done? Was it done in a similar setting or not?
2. Why the interview was not held in a separate room? Because, due to the fear of other people around the wards/interview areas, the participant may not give ample pieces of information which may hinder your findings. To elaborate more, there might be certain sensitive issues that might be positive or negative effects on the implementation of KMC. But, due to the fears of other caregivers other than the teams that participated in the data collection, the interviewed mothers/caregiver could be deterred from telling the truth, which may highly affect the result of your work. Please, consider this issue and amend it as much as possible.
3. When data saturation were determined? To clarify more, how many times the data were repeated to say the data were saturated? Please, make it clear!

**In the strength and limitations:**

1. One of the limitations of the study was, no follow-up study in hospital A, due to the outbreak of the COVID-19 pandemic. So, why not you consider other possible preventive strategies and continue the follow-up study? For example, by wearing a face mask you can continue the study as intended.
2. In general, what did you do to handle the impacts of the pandemic on your research findings? Please, incorporate as a strength of your study, if any activities were done to alleviate the impacts of the pandemic.
3. The other limitation of the study was, that there were two-three people in the room together with the caregiver during the time of the interview. So, why not you did the interview in a separate room or lonely? What were the challenges? Please, include the challenges, if any. If there were no challenges to doing the interview in a separate room or lonely, the way you did the interview was not scientifically acceptable.
