# Supplementary material for: Exploring caregivers’ experiences of Kangaroo Mother Care in Bangladesh: A descriptive qualitative study
Source: PLoS One. 2023 Jan 23;18(1):e0280254. doi: 10.1371/journal.pone.0280254 (PMC9870098; doi:10.1371/journal.pone.0280254)
Supplement: Attachment — Submitted filename: Response to reviewers.docx [file pone.0280254.s010.docx]

**Response to Reviewers**

The authors would like to thank the reviewers for their input and highly valuable comments for the article titled “Exploring caregivers´experiences of Kangaroo Mother Care in Bangladesh- a qualitative study”. We believe that the review has resulted in an improved manuscript. Point-to-point answers follow below.

Reviewer #3: I read the manuscript again in detail and the manuscript is well-structured and written in a good manner. The author addressed all my comments and I recommended to accept the paper.

Reviewer #4: Manuscript Number: PONE-D-22-08128R1
Manuscript Title: Exploring caregivers’ experiences of Kangaroo Mother Care in Bangladesh– a qualitative study.
Thank you very much, Dr. Tai-Heng Chen, Academic editor of the PLOS ONE journal for giving me this opportunity to review this scholarly work which was grounded on a very interesting topic “Exploring caregivers’ experiences of Kangaroo Mother Care in Bangladesh– a qualitative study”.
I would also appreciate the authors of this manuscript for investigating this very important topic and coming up with such interesting findings. I have been impressed with the flow, organization, and way they synthesize the research from beginning to end. All the parts of the manuscript have been clearly stated and narrated. I appreciate the authors for their devoted work. Having this in mind, I do have some questions, comments, and suggestions to be addressed.
I had put all this concerns previously in the "Upload reviewer attachement section", but it was not adderessed.

**General questions:**

1. What type of qualitative study it was? Was it phenomenological, Ethnographic, Grounded theory, or ……

*Thanks for the comment. The study is an explorative descriptive qualitative study. Now the title is changed to Exploring caregivers´s experiences of Kangaroo Mother Care in Bangladesh- a descriptive, qualitative study.*

2. What were the questions of your research?

*Thanks for the comment. The question of the research is stated as the objective of the study in the manuscript. The objective of this study was to explore caregivers' experiences of performing KMC in hospital settings and after continuation at home in Bangladesh, with the intention to identify enablers and barriers for optimal implementation.*

**In the methods section:**

In the data collection sub-section;
1. When, and where had the pilot study been done? Was it done in a similar setting or not?

*Thanks for the comment. The pilotinterview was performed in one ot the 2 study hospitals, the day before the first interview for the datacollection. The interview was not included in the study as privacy was not ensured during this interview. This has been clarified in the manuscript.*

2. Why the interview was not held in a separate room? Because, due to the fear of other people around the wards/interview areas, the participant may not give ample pieces of information which may hinder your findings. To elaborate more, there might be certain sensitive issues that might be positive or negative effects on the implementation of KMC. But, due to the fears of other caregivers other than the teams that participated in the data collection, the interviewed mothers/caregiver could be deterred from telling the truth, which may highly affect the result of your work. Please, consider this issue and amend it as much as possible.

*Thanks for the important comment. The children at the ward were in need of neonatal care and was in skin to skin position when the interview were conducted. Together with the healthcare staff it was decided that the child should not leave the ward, which is why the interviews were conducted in the ward. In order to ensure a safe environment for the interviews the nurse in the ward the healthcare staff was not present in the room at the time of the interview. In that way, privacy was ensured despite being held in the ward. The distance between the beds were also large enough to prevent the other people in the ward to overhear the conversation. In the second set of interviews, conducted at the one-week follow-up, the interviews were held in a separate room with no other patients or healthcare staff present. This has been clarified now in the manuscript.*

3. When data saturation were determined? To clarify more, how many times the data were repeated to say the data were saturated? Please, make it clear!

*We noticed the answers were similar for the in- ward interviews until PI saw the themes were repeated. Follow up interviews were finished earlier but when analyzing the result form the follow-up interviews we saw similar themes and therefor these interviews was considered to be saturated. This has been updated in the manuscript.*

**In the strength and limitations:**

1. One of the limitations of the study was, no follow-up study in hospital A, due to the outbreak of the COVID-19 pandemic. So, why not you consider other possible preventive strategies and continue the follow-up study? For example, by wearing a face mask you can continue the study as intended.

*Thanks for the comment.* *The clinic decided the interviews could not be continued due to the risk of spread of the coronavirus and the lock down in the whole country. When the society was adopted to digital solutions it was not feasible to continue the follow up interviews.*

2. In general, what did you do to handle the impacts of the pandemic on your research findings? Please, incorporate as a strength of your study, if any activities were done to alleviate the impacts of the pandemic.

*Thanks for the comment.* *Due to the sudden lock down in the country due to Corona we couldn’t continue the data collection for the follow-up interviews. During the analysis we found we got enough of data and saturation were fulfilled. This has been discussed in the manuscript.*

3. The other limitation of the study was, that there were two-three people in the room together with the caregiver during the time of the interview. So, why not you did the interview in a separate room or lonely? What were the challenges? Please, include the challenges, if any. If there were no challenges to doing the interview in a separate room or lonely, the way you did the interview was not scientifically acceptable.

*Thanks for the comment. Regarding the first point raises, notes were taken on the environment during the interviews by the first two authors, they did not speak Bangla and they were available to clarify questions if needed, to come up with follow-up questions to fully understand the experience and opinions of the caregivers, as well as to take notes on the interview environment. So therefor it was not only one person performing the interview.*

*Regarding the next point why the interview was not held in a separate room, please see the same response at point 2 under method section. The children at the ward were in need of neonatal care and supervision and was in skin to skin position when the interviews were conducted. Together with the healthcare staff it was decided that the child should not leave the ward, which is why the interviews were conducted in the ward ant not in a separate room. In order to ensure a safe environment for the interviews the healthcare staff was not present in the room at the time of the interview. In that way, privacy was ensured despite being held in the ward. The distance between the beds were also large enough to prevent the other people in the ward to overhear the conversation. In the second set of interviews, conducted at the one-week follow-up, the interviews were held in a separate room with no other patients or healthcare staff present. This has all been clarified in the manuscript.*
