# Supplementary material for: Exploring caregivers’ experiences of Kangaroo Mother Care in Bangladesh: A descriptive qualitative study
Source: PLoS One. 2023 Jan 23;18(1):e0280254. doi: 10.1371/journal.pone.0280254 (PMC9870098; doi:10.1371/journal.pone.0280254)
Supplement: Attachment — Submitted filename: Reviewer comments.docx [file pone.0280254.s007.docx]

Reviewer reports

Title: Exploring caregivers’ experiences of Kangaroo Mother Care in Bangladesh– a qualitative study

Reviewer: Mesfin Abebe

Thank you for the opportunity to review this manuscript I read the manuscript in detail and the manuscript is well-structured and written in a good manner. However, I raised some minor comments and suggestions below. I'm hopeful the comments below can help fill these gaps and improve the quality of the paper.

Minor revision

The paper's overall language quality is a little low; there are numerous grammatical errors throughout the entire manuscript. Before the next submission, I recommend that the entire manuscript be revised by a native English speaker.

Abstract: The abstract accurately reflects the details presented in the manuscript's body. However, the use of abbreviations in the abstract section is not recommended, and I recommend revising it.

Background: your background information is somewhat good, but didn’t show a gap in your study.

Methods:

1. Clearly state the sampling procedure you used to choose the 15 participants.

2. You only interview 15 mothers in order to answer your research question. Do you have saturation data based solely on interview methods of data collection?

3. Why were only semi-structured interviews considered?

4. What were the admission requirements? Was admission made immediately after birth, or was a period of stabilization in a special newborn care unit?

5. How long did it take from birth to admission to kangaroo care?

Results

1.	The result is generally well written and has good thematic contents

Discussion:

1.	The discussion is generally well-organized and incorporates existing literature. There might be more consideration given to the next steps in the research or implementation

2.	What do mean skin-to-skin sessions?

3.	Clear the phrase “out-of-pocket money”
